# Dual-fiber optical tweezers integrating high-sensitivity structured-light displacement measurement system on fiber end-face

**DOI:** 10.1038/s41598-025-93523-2

**Published:** 2025-03-17

**Authors:** Guofeng Li, Wei Xiong, Haining Feng, Zijian Feng, Tengfang Kuang, Zhechun Lu, Xiang Han, Xin He, Xinlin Chen, Junbo Yang, Guangzong Xiao

**Affiliations:** 1 College of Advanced Interdisciplinary Studies, National University of Defense Technology, Changsha, 410073 Hunan China; 2 Nanhu Laser Laboratory, National University of Defense Technology, Changsha, 410073 Hunan China; 3 Center of Material Science, National University of Defense Technology, Changsha, 410073 Hunan China

**Keywords:** Dual-fiber optical tweezers, Structured-light displacement method, High sensitivity, Miniaturization and integration, Optical manipulation and tweezers, Optical sensors

## Abstract

The dual-fiber optical tweezers have become widespread in trapping, assembling, and sensing due to their simple fabrication process and flexible operation. However, the miniaturization and integration of their displacement measurement optical paths remain challenging. Here, we propose and experimentally demonstrate an integration of structured-light displacement (SLD) measurement method tailored for dual-fiber optical tweezers. A key component split-waveplate is integrated onto the fiber end via coating and etching in the SLD method. The etched fiber and another single mode fiber form optical tweezers, which enables to trap particle and measure its position simultaneously without additional optics. More importantly, it demonstrates a superior signal-to-noise ratio after filtering out the trapping field by the etched fiber. Our results demonstrate a displacement sensitivity reaching the 0.1 pm/Hz^1/2^ level, which surpasses the performance of most results using the quadrant photodiode method. Ultimately, we discussed the possibilities of using two etched fibers to detect displacements in different directions, or integrating this method into a single optical fiber. This method has significant potential applications in precision sensing, contributes to the integration of optical tweezers and fosters the development of lab-on-fiber applications.

## Introduction

In 1970, Arthur Ashkin demonstrated the use of optical forces to manipulate the motion of microparticles^1^. This pioneering work evolved into optical tweezers technology, driving significant advancements in biology^2–4^, precision measurement^5–7^, quantum sensing^8,9^, and various other fields^10–14^. Although optical tweezers in vacuum offer unmatched precision in object manipulation and ultra-high sensitivity in detection, they present challenges such as complex optical path structures and the large volume of system. In 1993, Constable et al. proposed a promising scheme involving dual-fiber optical tweezers characterized by a compact structure, simple fabrication process and flexible operation^15^. This setup is not only compatible with chip devices^16^, but also exhibits versatile in functions, including optical stretchers^17,18^, optical rotators^19,20^, and optical binding^21^. With the advancement of integrated photonics, the innovative concept of lab-on-fiber has emerged, integrating multiple optical paths or devices within a single fiber^22,23^. As lab-on-fiber technology has advanced, an array of functionalities, including sensing^24,25^, trapping particles^26,27^, and manipulating light^28,29^, have been meticulously incorporated into the fiber platform, demonstrating its versatility and potential.

Most optical tweezers applications fundamentally rely on displacement measurements of the trapped object. The video-based position detection method offers the advantage of direct observation of the particle and is suitable for measuring low-frequency displacements^30,31^. High-precision displacement measurements primarily utilize the back focal plane method, which employs scattered light to extract the particle displacement information^8,32–34^. Among these methods, the quadrant photodiode (QPD) method and differential displacement measurement using D-shaped mirrors are the most commonly employed techniques^35,36^. Nevertheless, it’s difficult to miniaturizing such measurement methods to match the fiber optical tweezers. The challenge mainly arises from the necessity to preserve the mode shape of the scattered light. Several studies have attempted to integrate displacement measurement into fiber optical tweezers^37–39^. However, there are no highly sensitive displacement measurement technologies tailored for fiber optical tweezers. It has constrained the advancement of fiber optical tweezers for integrated quantum sensing. In 2021, Lars. S. Madsen et al. presented an ingenious structured-light detection (SLD) method^40^. This method filters the trapping mode by flipping its transmission phase, allowing it to measure the displacement of particles using light power directly. It provides an alternative for the miniaturization of displacement measurement in fiber optical tweezers.

In this paper, we simultaneously capture a microsphere and measure its displacement using an integrated structured-light displacement measurement method tailored for the dual-fiber optical tweezers. A critical component, the split-waveplate in the SLD method is integrated onto a fiber end-face through coating and etching. The light passing through the particle is collected by the etched fiber (EF) in the fiber optical tweezers, and the trapping field is partially filtered out. Consequently, this setup successfully achieves radial displacement detection with a superior signal-to-noise ratio. This work advances high-precision sensing technology and provides new insights into integrating optical tweezers. It enables the fabrication of MEMS devices capable of on-chip acceleration sensing^41,42^, non-Newtonian force detection^43^, and viscosity coefficient measurement^44^, thereby promoting the development of lab-on-fiber technology.

## Principle

In the field of optical tweezers, most trapping systems use fundamental Gaussian beams (TEM_00_) as the trapping light, except for specific applications that require special light fields. The trapping light is partially disturbed by the microsphere and converted into a new field that carries the information about the position of the particle, while the undisturbed trapping light remains as the fundamental mode TEM_00_. The undisturbed trapping light is symmetric about the trap axis and occupies the majority of the probe beam. This part of light carries no displacement information and can serve as background. The information-containing field can be approximately described as an antisymmetric TEM_01_ mode, but it only constitutes a small portion of the probe beam^34^. In the SLD method, a split-waveplate is crucial for filtering the trapping field before coupling the probe beam into the single mode fiber (SMF)^40^. The split-waveplate is specially designed to introduce a π-phase shift to the light on one half of the field, while leaving field on the other half unchanged. The phase difference between the two halves reverses the symmetry of two modes in the probe beam, converting the TEM_00_ mode into an antisymmetric mode and the TEM_01_ mode into a symmetric flipped TEM_01_​ mode. The flipped trapping field become a high-order mode and cannot propagate through the fiber. In comparison, the flipped TEM_01_​ mode shows a limited overlap in amplitude and phase within certain regions with the fundamental mode, enabling that to couple into the SMF^45,46^. In a word, the split-waveplate combined with single mode fiber (SMF) acts as a spatial filter to diminish the trapping field and enhance the transmission of the information-containing field, thereby realizing high signal-to-noise ratio displacement detection.

**Fig. 1:**
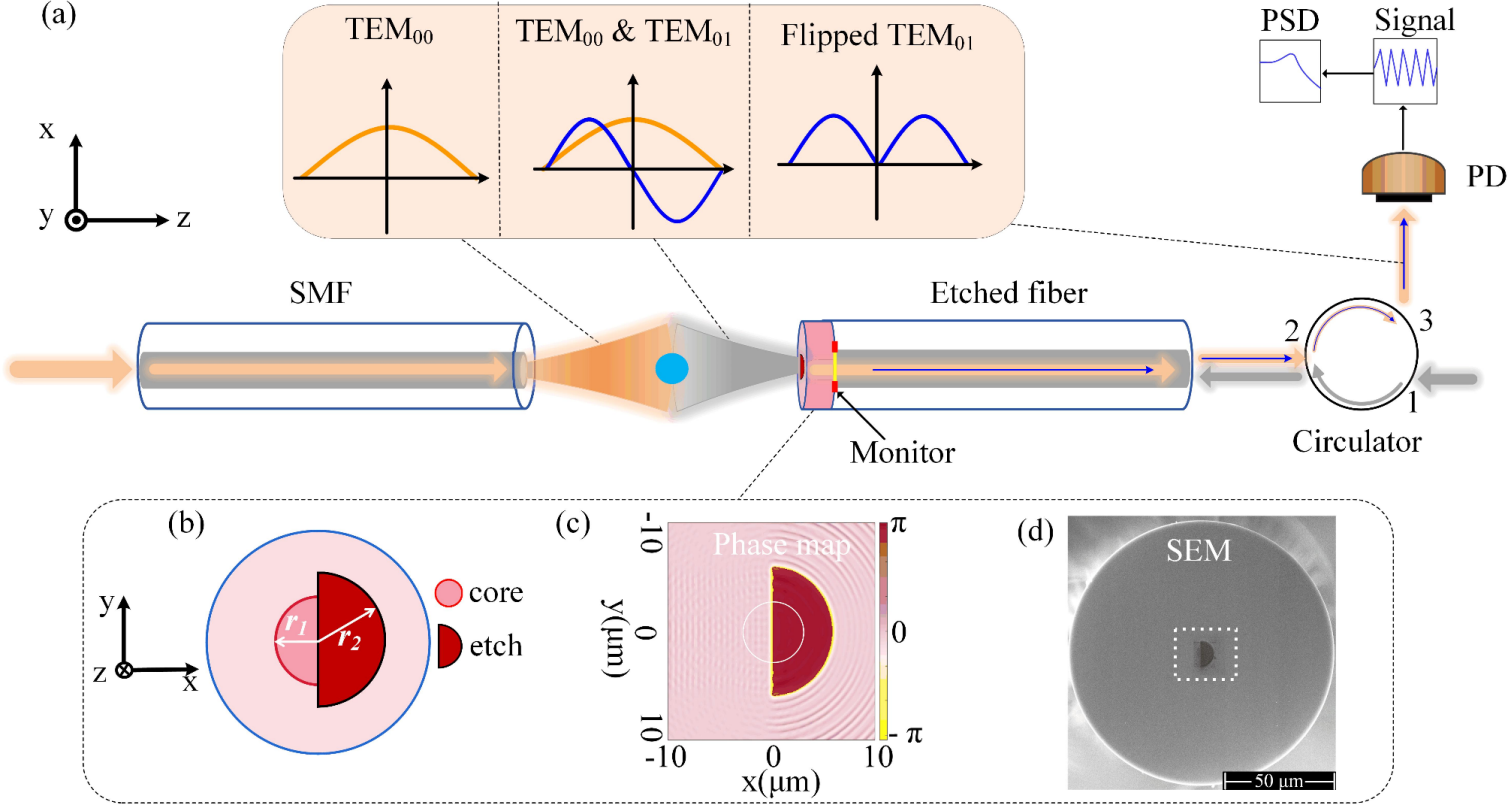
Principle of the integrated structured-light displacement measurement method tailored for dual-fiber optical tweezers. (**a**) Schematic representation of dual-fiber optical tweezers composed of SMF and etched fiber. The end face of the etched fiber is coated with a Ta_2_O_5_ film (pink layer). PD indicates that the photodetector is used to convert optical signals into electrical signals. Power spectral density (PSD) is obtained by Fourier transform. Orange inset: the TEM_00_ mode is the trapping field, and the TEM_01_ mode indicates the information-containing field. (**b**) Schematic of the etched fiber end face with etching structure (crimson), *r*_1_ is the radius of the fiber core, and *r*_2_ is the radius of the etched semicircle. (**c**) Simulated phase map of etched fiber, showing a phase difference π between the etched and the unetched region. (**d**) SEM image of the fiber with an etched semicircular structure in a white dotted box.

Figure 1(a) depicts the schematic of the integration of structured-light displacement measurement method tailored for dual-fiber optical tweezers. The etched fiber and another SMF form a dual-fiber optical tweezer to trap particles. The etched fiber is fabricated in two steps. First, a standard SMF is coated with a Ta_2_O_5_ layer (depicted as the pink layer in the figure)^47^. Then, the coating layer is etched to a certain depth using a Focused Ion Beam (FIB). As a result, a phase difference of π is generated between the etched and unetched regions on the end face of the fiber due to the optical path difference. As the trapping light (TEM_00_ mode) transmits through the trapped particle, the perturbative caused by the particle transforms a portion of the light into the TEM_01_ mode (the blue curve in the orange inset)^40^. When the light passes through the coating layer with an etching structure, the symmetries of the two modes are reversed. Consequently, the antisymmetric trapping field cannot propagate in the single-mode fiber portion of the etched fiber. In this configuration, the probe beam received by the etched fiber enters the circulator through port 2. Then, it transmits via port 3 to a photodetector (PD). As a result, the PD predominantly detect the flipped TEM_01_ mode, thereby extracting the displacement information of the microsphere. This configuration significantly enhances the signal-to-noise ratio for displacement measurements by utilizing mode transformation to maximize the transmission of the displacement signal.

Figure 1(b) is the schematic of the etched fiber end-face with etching structure, where *r*_1_ is the radius of the fiber core and *r*_2_ indicates the radius of the etched area (crimson semi-circle). In the simulation, the monitor is positioned between the coating layer and the optical fiber. As shown in Fig. 1(c), the phase map clearly indicates a π phase difference between the etched and unetched regions. This phase difference is realized by the optical path difference during the transmission of light in the film layer. Figure 1(d) demonstrates Scanning Electron Microscopy images (SEM) of the end face of the etched fiber. An etching semicircle within the white dotted box has a depth equal to the film thickness.

We utilized the finite difference time domain (FDTD) method to build the simulation model. Both simulations and experiments were conducted in a water environment. The fiber (Corning, HI 1060) with a core radius of *r*_1_ 2.65 μm and a mode-field diameter of 5.9 ± 0.3 μm @980 nm. To match the two-dimensional profile size of the beam emitted from the SMF to the dimensions of the etched semicircle in the fiber coating layer. It enables to introduce a phase difference of π between the etched and unetched regions. Consequently, the radius *r*_2_ of the etched semicircle is precisely selected to be 6 μm.

**Fig. 2:**
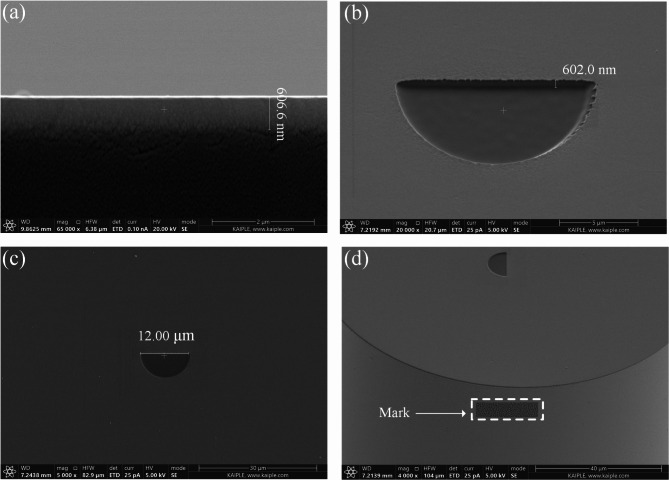
SEM image of the Etched fiber. (**a**) Sideview of the Ta_2_O_5_ layer. (**b**) Enlarged image of the etched semicircular. A tilt angle is set between the fiber end face and the horizontal plane to facilitate measurement of the etching depth. (**c**) Top view of the fiber end face. (**d**) Marking on the lateral aspect of the fiber.

The transmission phase formula is as follows: ${\text{\varvec{\Delta}}}\varphi \cdot ={\text{\varvec{\Delta}}}n{k_0}d$, where ${k_0}=2\uppi /\lambda$ is the free space wave vector, *n* is the refractive index difference of the uniform medium, and *d* is its thickness. The thickness of the Ta_2_O_5_ layer in fiber end face is designed as 600 nm. We deposited Ta_2_O_5_ films on the fiber end-face using ion sputtering technology, achieving a thickness of 606.0 nm, as shown in Fig. 2(a). The ideal refractive index of Ta_2_O_5_ at a wavelength of 980 nm is 2.157, while the experimentally measured refractive index, determined using an ellipsometer, is slightly lower at *n*_1_ = 2.155. The depth of the etched semicircle is the same as the thickness of the film, as shown in Fig. 2(b). The refractive index of water is *n*_2_ = 1.33, thus the phase difference between the etched and non-etched parts is 1.01π. In this way, approximately π phase is achieved. It satisfies the phase requirements of the structured-light displacement measurement method for flipping mode. Figure 2(c) presents the diameter of the etched semicircle, while Fig. 2(d) displays the mark on the lateral aspect of the etched fiber. This mark is designed to locate the fiber direction during the subsequent assembly of dual-fiber optical tweezers. It is worth noting that before carrying out the experiment, the gold film sprayed by the FIB processing needs to be removed using aqua regia to avoid affecting the transmission phase.

## Result and analysis

### Optical trap

We calculate the axial forces on the microspheres at different distances (from 120 μm to 40 μm) between two optical fibers. Results indicate that at an output power of 100 mW for each fiber, a point where the axial force is 0 pN with a negative slope appears when the distance is reduced to approximately 40 μm. It means that the microsphere is subjected to equal force from the dual beams and in a stable trapped state. If a larger fiber spacing is chosen, the light power emitted from the etched fiber needs to be increased. However, there is a concern that under high-power output in a liquid environment, the liquid could be heated and may cause bubble formation. Consequently, we chose a fiber spacing of 40 μm between the two fibers to set up the dual-fiber optical tweezers.

**Fig. 3:**
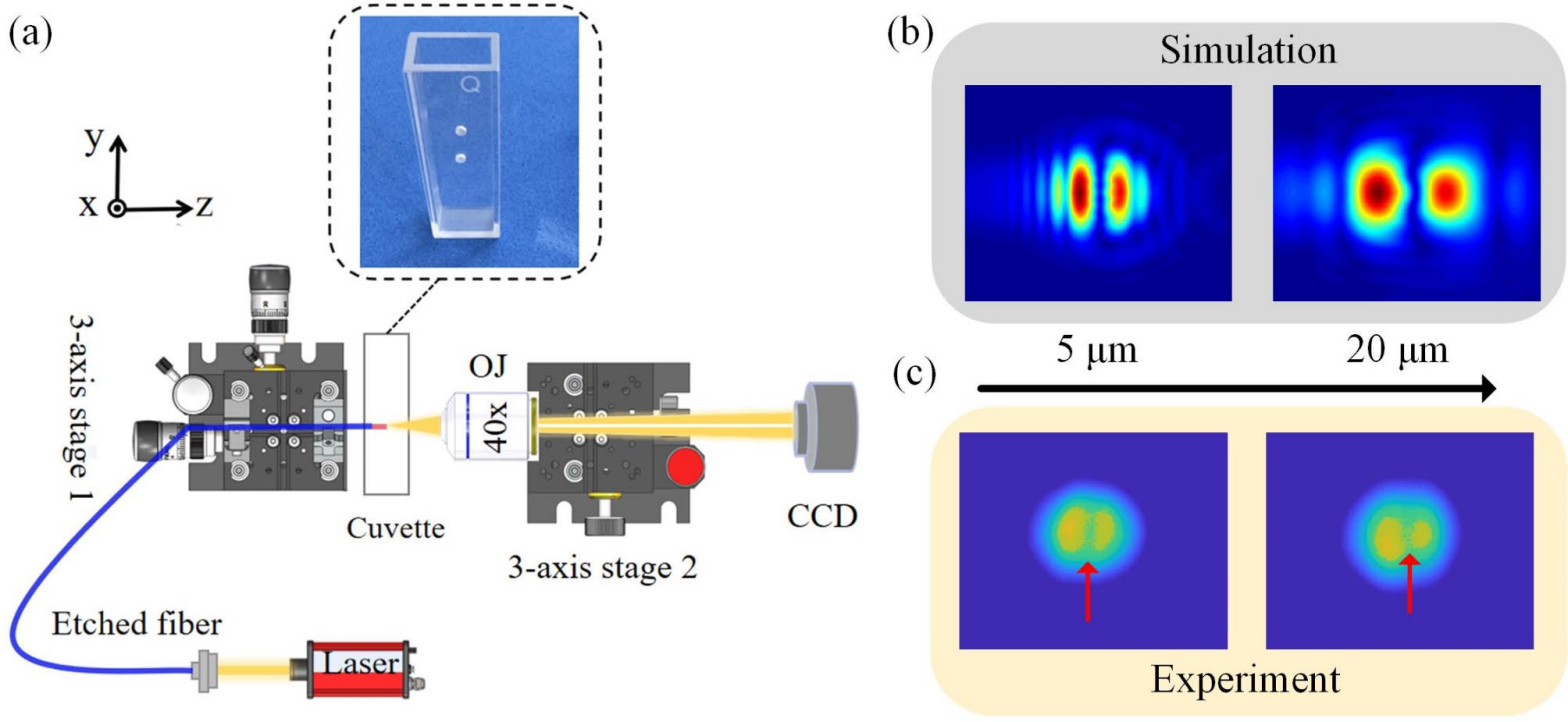
(**a**) Schematic diagram of the optical fiber output mode field testing system, and the cuvette in the illustration features two coaxial micropores; (**b**) The intensity distribution of the etched fiber beam at different transmission distances is obtained by simulation (**c**) The intensity distribution of the etched fiber beam at different transmission distances is obtained by experiment.

Figure 3(a) shows the schematic diagram of a beam testing system. The cuvette in the illustration features two coaxial holes. The etched fiber extends through the hole into the water-filled cuvette during testing. When the focus of the objective lens coincides with the end face of etched fiber, the measured beam mode field diameter is minimized, marked as z = 0 μm. By moving translation stage 1 along z axis, the beam intensity distribution at different transmission distances can be measured.

Figure 3(b) presents the simulated intensity distribution of the beam emitted from the etched fiber, and it exhibits a bimodal distribution with two distinct peaks of similar intensity. In the FDTD simulations, the amplitude of the light source was set to 1, and the polarization aligned along the x-axis. The electric field strength distribution was normalized. Consequently, the intensity plots of simulation are on the same scale. In the actual process of etched fiber, it is difficult to ensure that the etching demarcation line is entirely centered on the fiber core. Hence we have introduced a 300 nm offset of the semicircle from the central position of the fiber core in the simulations.

In the experiments, we coupled white light into the other end of the etched fiber. Under a microscope, we excluded samples with excessive offsets of the etched boundary from the fiber core center. Using the testing setup in Fig. 3(a), the intensity distribution of the beam emitted from the etched fiber at different transmission distances was measured, as shown in Fig. 3(c). A dark fringe appears at the center of the beam intensity distribution, as indicated by the direction of the red arrow, which is due to destructive interference caused by the π phase difference between the etched and unetched regions. These results closely align with the simulation intensity distribution, demonstrating a consistent trend. It is expected that the microsphere near the optical axis will interact less strongly with the light emitted by the etched fiber, given the split nature of its spot. To effectively trap a particle near the central position between the two fibers, it is necessary to increase the output power of the etched fiber.

**Fig. 4:**
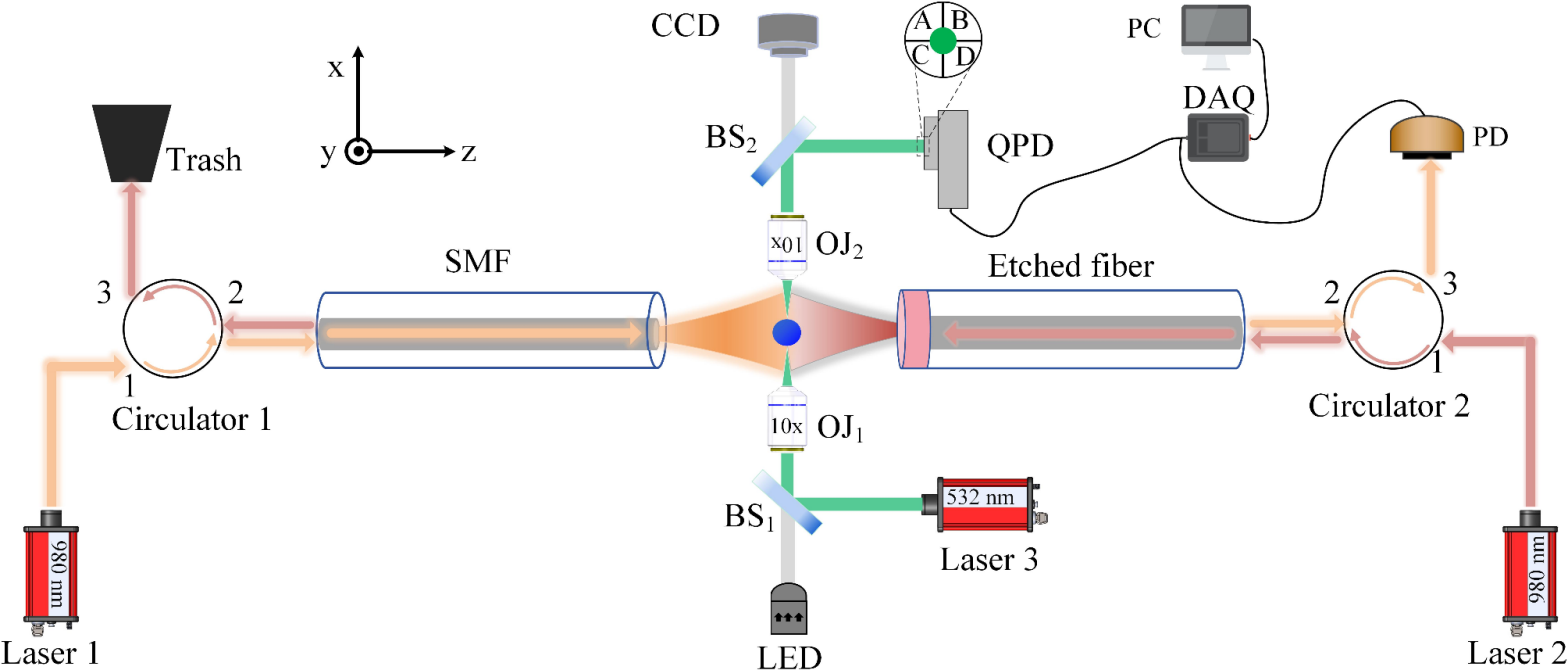
The dual-fiber optical tweezers system with the SLD method and the QPD method. PD stands for Photodetector. QPD, the quadrant photodiode. LED, light emitting diode. CCD, Charge coupled Device. OJ1 & OJ2, objective. BS1 & BS2, beam splitter. DAQ, data-acquisition card. PC, computer. Trash, optical trash can.

Figure 4 shows the dual-fiber optical tweezers system with the integrated structured-light displacement method and the QPD method. A LED light and a CCD camera form an illumination imaging optical path for observing trapped particle. The light (orange) input by laser 1 enters the SMF through the circulator 1, passes through the particle and couples into the etched fiber, and is finally received by the PD. In order to facilitate light path adjustment, the laser wavelength used in the QPD method is 532 nm (green). The detection beam of the QPD method is collected by objective 2 and finally enters the QPD. The PD and QPD are connected to the DAQ card to store data on a computer. The sampling frequencies of the SLD method and QPD method are 1000 kS/s and 200 kS/s, respectively.

**Fig. 5:**
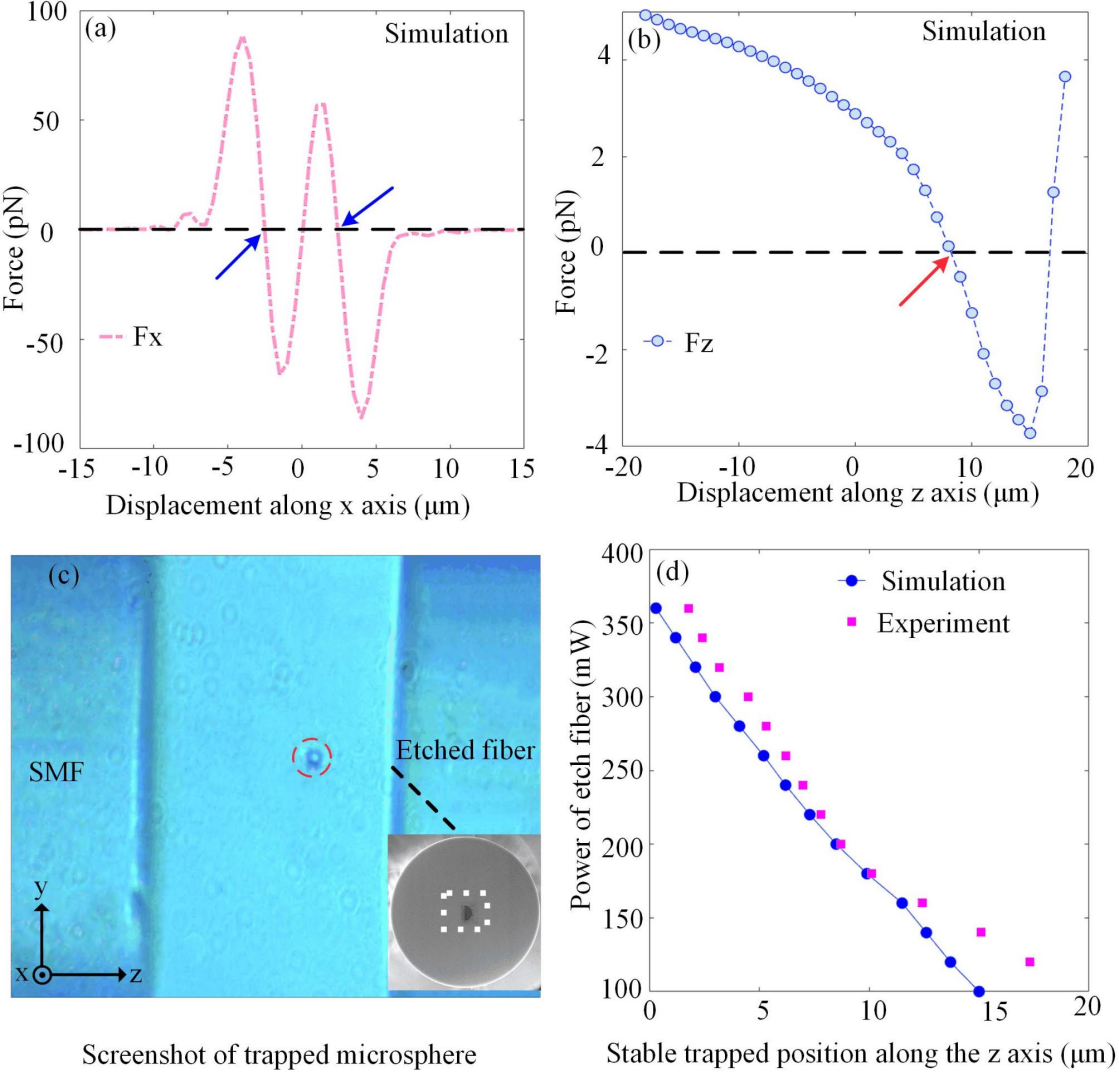
Calculation of optical forces and capture of microsphere with a diameter of 5 μm. (**a**) The radial force on microsphere. (**b**) The axial force on microsphere. (**c**) Screenshot of a silica microsphere (red dotted circle) trapped by the dual-fiber optical tweezer, with the inset displaying the SEM image of the etched fiber’s end face. (**d**) Relationship between the equilibrium position of the microsphere and the input power of the etched fiber (*P*_2_).

Based on the calculations from the previous section, we set the power output of the SMF (*P*_1_) to 100 mW and that of the etched fiber (*P*_2_) to 200 mW. The simulation results for the radial force on a silica microsphere with a diameter of 5 μm is presented in Fig. 5(a), two points with zero net force and negative slope (blue arrow) indicates the existence of two stable trapping points in the radial direction. The two equilibrium points are similarly distant from the central axis with similar slopes, suggesting that the axial optical force and displacement response of the particle are essentially the same. The corresponding radial force curve is depicted in Fig. 5(b). An axial equilibrium point (red arrow) is observed near the central region between two fibers. These simulation results confirm that our dual-fiber tweezers can stably capture particles. We performed experiments to successfully trap a silica microsphere with a diameter of 5 μm, and its screenshot is provided in Fig. 5(c). In liquid environments, measuring the optical power output from fibers poses significant challenges. However, due to the inherently low loss of single-mode fibers, the discrepancy between input and output power is minimal. Consequently, using the input power for experiments can ensure both accuracy and repeatability. Figure 5(d) illustrates the relationship between the input power of the etched fiber and the equilibrium position of the microsphere. It is observed that as *P*_2_ increases, the microsphere gradually moves from a position which close to the etched fiber towards the center. The experimental data align with the simulated trends, thereby validating the accuracy of the simulation.

### Displacement measurement

**Fig. 6:**
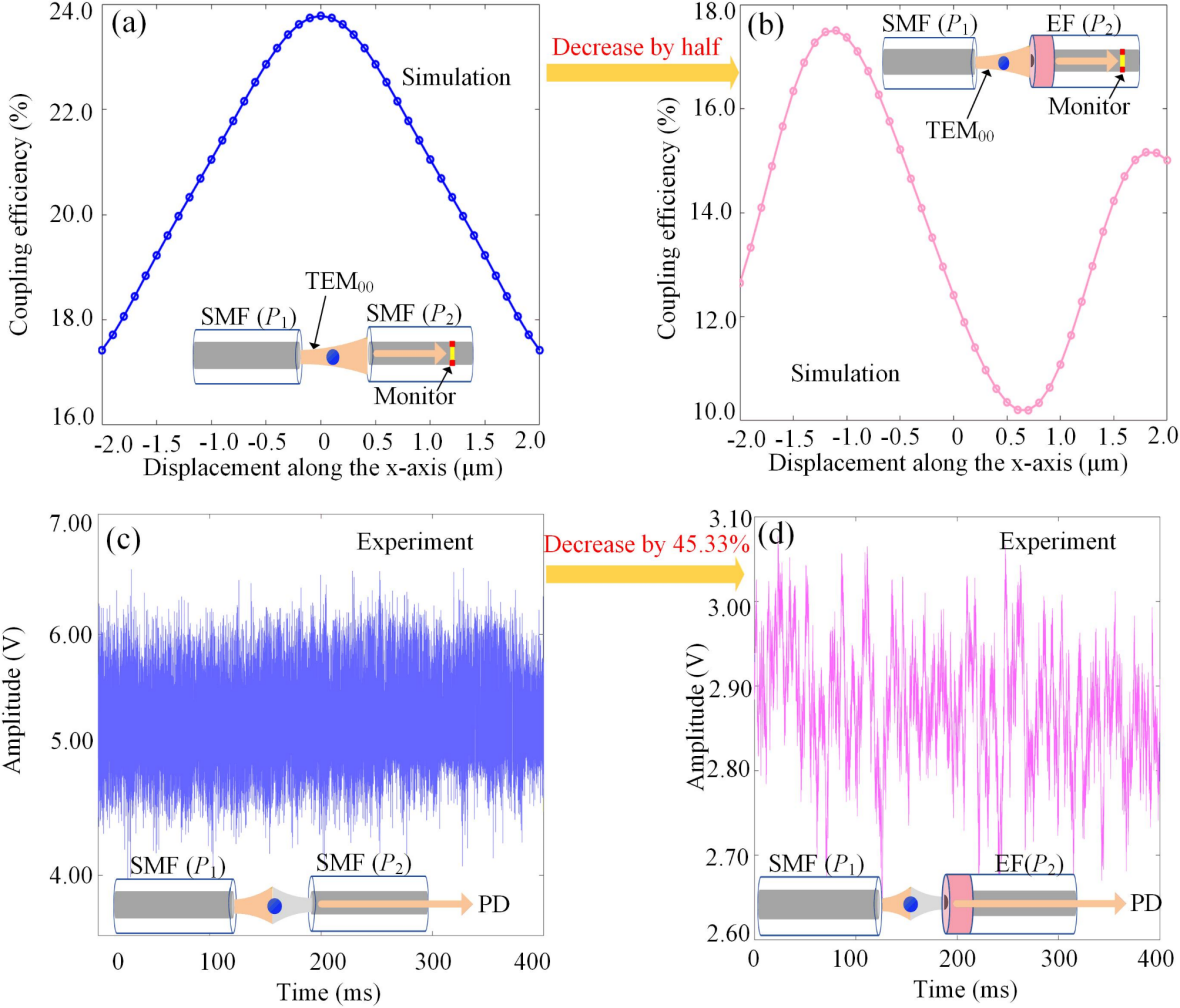
The filtering effect of the etched fiber on the trapping field. (**a**), (**b**) Coupling efficiency of the SMF-Etched fiber (EF) and the SMF-SMF optical tweezers. (**c**), (**d**) The voltage amplitudes of the signal received by the PD in the SMF-SMF and the SMF- EF optical tweezers.

In the dual-fiber optical tweezers, light from a SMF passes through the microsphere and couples into the other fiber. When light transmit to the EF, the etched fiber tip flips the phase of half the field, reversing the symmetry. As mentioned in the principle, the symmetric component will be suppressed and the antisymmetric component carrying displacement information will be retained. The fiber coupling efficiency can be used to determine the information collection capability. The coupling efficiency of the SMF-SMF and SMF-EF configuration are simulated and depicted in Fig. 6(a) and (b), respectively. In Fig. 6(a), the coupling efficiency follows a symmetrical parabolic distribution with the displacement of the trapped microsphere. The largest coupling efficiency is approximately 24% when the microsphere locates on the optical axis (*x* = 0). In comparison, the coupling efficiency of the SMF-EF configuration is reduced to about 12% when *x* = 0, representing a half decrease compared to the SMF-SMF configuration [see Fig. 6(b)]. This reduction in coupling efficiency is mainly attributed to the filtering of the symmetric component which is regarded as the background. It’s beneficial for improving SNR of the displacement measurement. More importantly, the coupling efficiency is proportional to the displacement of the microsphere near *x* = 0, due to an offset of the etched semicircle. This provides a linear response region for displacement measurement and orientation determination of moving microspheres.

For the construction of dual-fiber optical tweezers, precise alignment of the two fibers was achieved by engraving V-grooves on an acrylic plate^48^. Initially, we constructed an optical tweezers system with a separation of 40 μm using two single-mode fibers [inset of Fig. 6(c)]. To mitigate thermal effects and ensure the stability of the trapped microsphere, the input power of the etched fiber was reduced. This adjustment also enabled long-term experimentation. Consequently, the input powers *P*_1_ and *P*_2_ of the two fibers were set to 100 mW and 200 mW, respectively. Subsequently, a silica microsphere with a diameter of 5 μm was successfully trapped. The data were collected by a data-acquisition card controlled by a LabVIEW program. The corresponding voltage signal is presented in Fig. 6(c), showing an average signal amplitude of 5.25 V. Under identical experimental conditions, we established an optical tweezers using a SMF and an etched fiber (inset of Fig. 6d). The input power from the SMF *P*_*1*_ and from the etched fiber *P*_*2*_ were maintained at 100 mW and 200 mW respectively. The photodetector (PD) connected to the etched fiber recorded an average voltage of 2.87 V. Compared to the SMF-SMF optical tweezers, the coupling power is reduced by 45.33% with the same trapped optical power. The experimental results correspond well with the simulation results. These results suggest that the trapping field was significantly filtered, which is conducive to the extraction of the information-containing field.

**Fig. 7:**
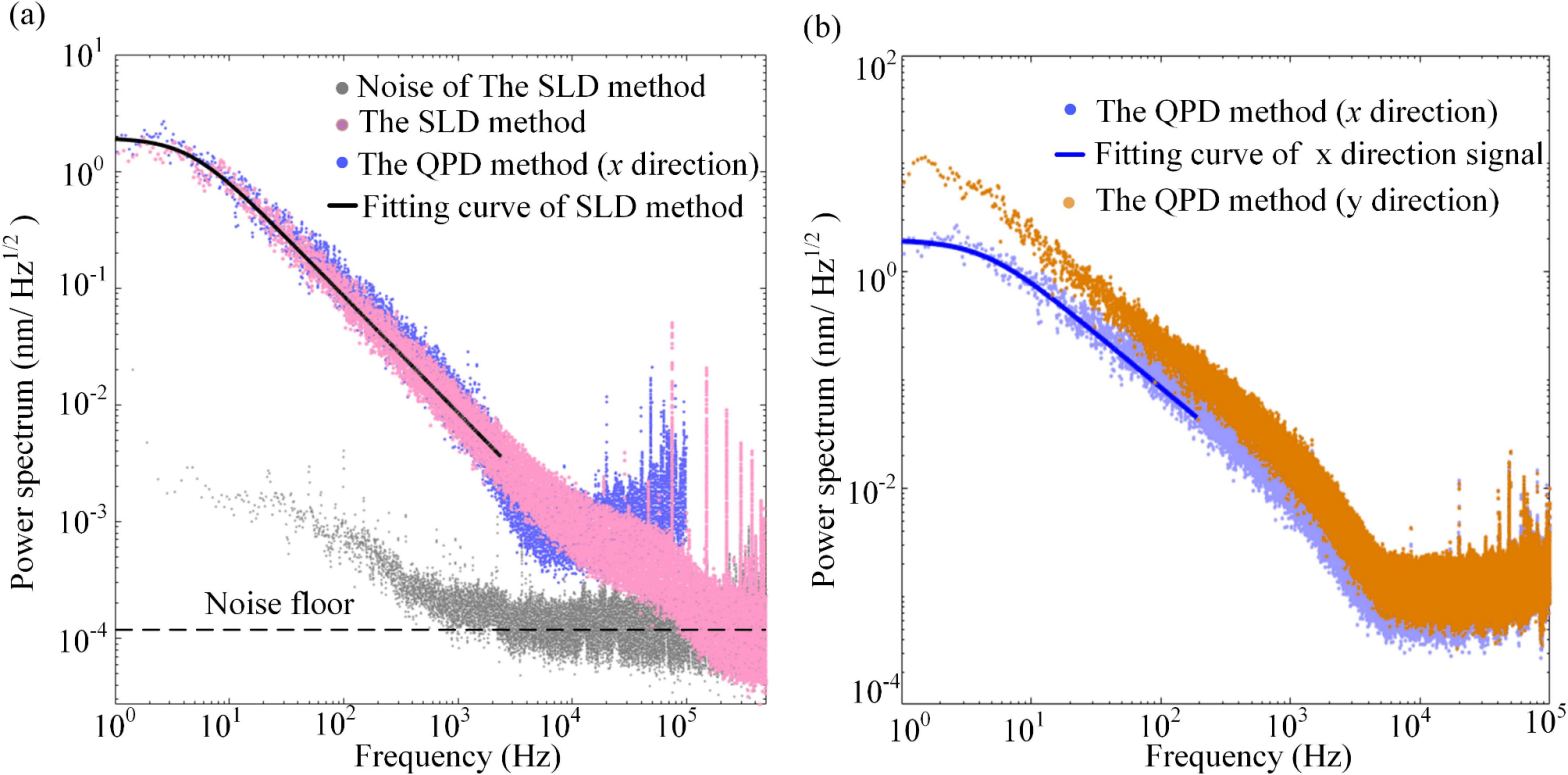
Power spectral densities a of 5 μm silica microsphere and noise. (**a**) The pink spectrum represents the power spectral density measured by the SLD method, and the solid black line is its Lorentz fitting curve. The gray spectrum represents the measurement noise of this method. The blue spectrum represents the power spectral density measured by the QPD method (x direction signal). (**b**) The blue spectrum represents the displacement spectrum in the x-direction measured by the QPD method, which is the same as the spectrum line in (**a**). The solid blue line is its Lorentz fitting curve. The orange spectrum represents the displacement spectrum in the x-direction measured by the QPD method.

Figure 7(a) presents the power spectrum density of experimentally recorded displacement of the trapped particle (the pink dots). The fitting curve (black solid line) was derived using the Lorentz fitting method^49^. The corner frequency of the displacement spectrum is *f*_*c*1_ = 3.21 Hz, and the trapping stiffness *k*_*x*1_ in the *x* direction was calculated to be 1.05 pN/µm. For comparison, another displacement measurement optical path was established using the QPD method. The displacement spectrum corresponding to the x direction signal measured by the QPD method is shown in Fig. 7(a) and (b). The blue solid line in Fig. 7. (b) is its Lorentz fitting curve, which has a corner frequency *f*_*c*2_ = 3.34 Hz, indicating a trapping stiffness *k*_*x*2_ of 1.09 pN/um. The spectra of the QPD and SLD methods overlap almost entirely in the low-frequency range up to 10^4^ Hz. The displacement spectrum of the y direction signal measured by the QPD method (in orange) is plotted in Fig. 7. (b). It can be observed that there is a significant difference compared to the displacement spectrum of the x direction in the low-frequency region. These results indicate that the SLD method can accurately measure the *x* direction displacement of microsphere. Moreover, the gray curve in Fig. 7(a) represents the signal PSD at the same optical power without the microsphere. This PSD indicates that the displacement detection sensitivity of the SLD method reaches 0.13 pm/Hz^1/2^, with a frequency range of 1–500 kHz. It is approximately half an order of magnitude higher than the QPD method in this experiment. Although using a higher sampling rate in the QPD method might offer comparable detection sensitivity, the advantage of the SLD method is that it can filter out the trapping field without attenuating the information-containing field.

Compared with the conventional displacement measurement method in optical tweezers^5,35,36,43,50^, this work achieves comparable sensitivity. Besides, using photodetector instead of QPD or balanced photodiode, this study can easily achieve MHz- to GHz-bandwidth. This high bandwidth is useful in the study of fast single-molecule dynamics and stochastic dynamics of Brownian motion^51,52^. Although some interferometry-based sensing methods can also reach high-speed detection, they are mainly sensitive to displacement along the optic axis, and a local oscillator is essential which makes the system more complex^53–55^. The trapping structure and detection system in this work are all made up of commercial fiber components. It is beneficial to realize the miniaturization of the optical trap. However, the cost of the EF is relatively high due to the complex fabrication process, this problem is expected to be solved by batch manufacturing of that.

## Summary and outlook

We have demonstrated the integration of a structured-light displacement measurement method for dual-fiber optical tweezers. The method enables the optical tweezers to simultaneously trap a particle and measure its displacement without additional optics. A split-waveplate was integrated onto the end-face of fiber through coating and etching. Unlike standard SMF, simulation and experiment reveal that the beam profile of the etched fiber divided into two parts. Based on optical force calculations, we selected a 40 μm fiber spacing to construct a dual-fiber optical tweezer, and successfully trapped a microsphere in experiments. In addition, we confirmed the effectiveness of the proposed method in the enhancing information-containing field. This method achieves a displacement measurement sensitivity of 0.1 pm/Hz^1/2^ level, surpassing the QPD method under comparable experimental conditions. With this research integrating structured light displacement detection into the fiber end face, some limitations still remain, such as the lack of multi-directional displacement detection capabilities.

We propose a conceptual design for multi-directional displacement detection using a dual-fiber optical tweezer, aiming to overcome the limitations of single-direction detection in conventional systems. This design utilizes two etched optical fibers with their etching boundaries oriented orthogonally to each other—one aligned perpendicular to the x-direction and the other perpendicular to the y-direction. By separately detecting the scattered light signals through these two etched fibers, displacement measurements along the x and y directions can be independently achieved. This concept establishes a framework for multi-directional displacement detection within dual-fiber optical tweezers, offering potential applications in microfluidics and precision optical sensing. However, practical implementation requires not only introducing an appropriate phase difference at the fiber end face but also optimizing the design structure to enhance the quality of the outgoing beam.

Inspired by the polishing processes of the side of a single-mode fiber^56–58^, it’s also possible to integrate this detection scheme into a single fiber. The fiber can be etched beyond the core region, and the cross-section at one end can be modified to introduce a phase difference of π. This approach enables simultaneous particle trapping and displacement measurement, significantly reducing system volume while improving integration and functionality.

Finally, we propose the potential for achieving multi-directional displacement measurements using two etched optical fibers, or by integrating structured-light displacement detection within a single fiber. The feasibility of these approaches for advancing lab-on-fiber systems was highlighted. Although there are still limitations regarding system volume and the implementation of multi-directional displacement detection, this study provides new perspectives for the development of lab-on-fiber technology and lays the foundation for future research in optical tweezers technology.

## Data Availability

The data used and analyzed during the current study are available from the corresponding author on reasonable request.
